# Enhancing the accuracy of HMM-based conserved pathway prediction using global correspondence scores

**DOI:** 10.1186/1471-2105-12-S10-S6

**Published:** 2011-10-18

**Authors:** Xiaoning Qian, Sayed Mohammad Ebrahim Sahraeian, Byung-Jun Yoon

**Affiliations:** 1 Department of Computer Science and Engineering, University of South Florida, Tampa, FL, 33620, USA; 2 Department of Electrical and Computer Engineering, Texas A&M University, College Station, TX, 77843, USA

## Abstract

**Background:**

Comparative network analysis aims to identify common subnetworks in biological networks. It can facilitate the prediction of conserved functional modules across different species and provide deep insights into their underlying regulatory mechanisms. Recently, it has been shown that hidden Markov models (HMMs) can provide a flexible and computationally efficient framework for modeling and comparing biological networks.

**Results:**

In this work, we show that using global correspondence scores between molecules can improve the accuracy of the HMM-based network alignment results. The global correspondence scores are computed by performing a semi-Markov random walk on the networks to be compared. The resulting score naturally integrates the sequence similarity between molecules and the topological similarity between their molecular interactions, thereby providing a more effective measure for estimating the functional similarity between molecules. By incorporating the global correspondence scores, instead of relying on sequence similarity or functional annotation scores used by previous approaches, our HMM-based network alignment method can identify conserved subnetworks that are functionally more coherent.

**Conclusions:**

Performance analysis based on synthetic and microbial networks demonstrates that the proposed network alignment strategy significantly improves the robustness and specificity of the predicted alignment results, in terms of conserved functional similarity measured based on KEGG ortholog (KO) groups. These results clearly show that the HMM-based network alignment framework using global correspondence scores can effectively find conserved biological pathways and has the potential to be used for automatic functional annotation of biomolecules.

## Background

With the increasingly high coverage of molecular interactions owing to the advancement of high-throughput techniques for measuring biomolecular interactions, such as the two-hybrid screening [1] and co-immunoprecipitation [2], comparative analysis of biological networks has recently attracted significant research attention. It has been demonstrated that comparative network analysis can provide an effective means of systematically studying molecular interactions in various organisms and gaining novel system-level insights [3-18]. For example, local network alignment across different species can identify similar subnetwork regions in the respective networks, which may lead to the discovery of conserved pathways that carry out essential cellular functionalities [3,5,6,9,11,15,16,19]. The concept of comparative network analysis can lead to the development of novel computational tools that allow us to transfer biological knowledge across species, especially from well-studied species to less-studied species [19].

Current local network algorithms [3,5,6,9,15] search for similar subnetwork regions by optimizing a pre-defined alignment score that incorporates the *topological similarity* of the interaction patterns in the compared networks as well as the *node similarity* of the molecules that belong to different networks, typically measured based on sequence similarity. To obtain better alignment results that are biologically more significant, there have been research efforts to improve the scoring scheme by incorporating evolutionary [4] or functional relationships [11,16] between molecules. Although there are various approaches for measuring the similarity between network nodes, most of the existing approaches compute this similarity based on the properties of individual nodes, such as their composition, functionality, or evolutionary relationships. However, cellular functions are carried out by collaborative efforts among many molecules, where interacting molecules may carry similar functionalities and share common characteristics. Therefore it would be reasonable to expect that, when evaluating the node similarity, incorporating additional information about the interacting molecules would enhance the network alignment results and lead to predictions that are biologically more meaningful.

Recently, we have introduced an effective framework for local network alignment based on hidden Markov models (HMMs), in which we integrate both the node sequence similarity and the interaction reliability into the scoring scheme by determining the parameters of the HMMs correspondingly [15]. We also developed an efficient dynamic programming algorithm that can find the closest pair of pathways from the respective networks in polynomial time. The HMM-based local alignment method can deal with a large class of path isomorphism and it allows one to search for long conserved pathways across large-scale networks. In this paper, we implement a semi-Markov random walk framework that diffuses the relationships of all the molecule pairs across the networks to obtain a *global correspondence score* between every pair of nodes. The resulting global correspondence score reflects the global similarity between nodes in different networks, by seamlessly integrating the topological similarity and individual node similarity. Alignment results based on synthetic networks and microbial protein-protein interaction (PPI) networks show that the performance of the HMM-based local alignment scheme can be significantly improved by utilizing the *global* correspondence score instead of the original *individual* sequence similarity score. The major contributions of this paper include the following: first, we integrate the global node correspondence scoring scheme into the HMM-based local network alignment framework [15], which leads to more accurate and robust alignment results; second, we thoroughly evaluate the performance of the proposed scheme based on synthetic benchmark networks, as well as real microbial networks, which clearly demonstrates the advantages of utilizing global correspondence scores, especially, in combination with the HMM-based framework.

## Methods

### Local network alignment based on hidden Markov models

In this section, we briefly review our local network alignment algorithm based on hidden Markov models (HMMs) [14,15]. We focus on aligning two biological networks to identify the common pathways that are conserved in both networks. Suppose we have two biological networks, represented as two graphs  and . In graph  of *N*_1_ nodes represents the corresponding molecules, and  of *M*_1_ edges indicates the presence of interactions *d_ij_* between the two molecules *u_i_* and *u_j_.* Similarly, we assume that  has a set  of *N*_2_ nodes and a set  of *M*_2_ edges. We denote the interaction reliability score between *u_i_* and *u_j_* in  as *w*_1_(*u_i_*, *u_j_*) and the interaction reliability between *v_i_* and *v_j_* in  as *w*_2_(*v_i_*, *v_j_*). The node similarity between  and  is denoted as *s*(*u_i_*, *v_j_*)*.*

In order to use HMMs to search for the pathways that are conserved in both networks, we search for the best matching pair of paths  and  of length *L* in the respective networks that maximizes the pathway alignment score *H*(**u**, **v**). The alignment score *H*(**u**, **v**) integrates the *node similarity score s*(*u_i_*,*v_j_*) between the aligned nodes *u_i_* and *v_j_* (1 ≤ *i*, *j* ≤ *L*), the *interaction reliability score w*_1_(*u_i_*, *u_i_*_+1_) between *u_i_* and *u_i_*_+1_ (1 ≤ *i* ≤ *L* – 1), the interaction reliability score *w*_2_(*v_j_*, *v_j_*_+1_) between *v_j_* and *v_j_*_+1_ (1 ≤ *j* ≤ *L* – 1), and the penalty for potential gaps in the alignment.

We first construct two HMMs respectively for two given networks. For , we design the state transition diagram of its corresponding HMM based on the graph structure of *.* The resulting HMM contains a hidden state for each node , which we also denote as *u_i_* for convenience. State transition is allowed from *u_i_* to *u_j_* for (*u_i_*, *u_j_*) such that *.* The HMM for  can be constructed in a similar way. To allow flexible node insertions and/or deletions in the alignment result, we add auxiliary states to the HMMs as described in [14,15]. The state transition probabilities of the HMMs are determined based on the interaction reliability scores *w*_1_(*u_i_*, *u_i_*_+1_) and *w*_2_(*v_j_*, *v_j_*_+1_). By introducing a “virtual observation sequence” **q** = *q*_1_ ⋯ *q_L_* that is *jointly* emitted by the two HMMs, we design the emission probabilities based on the node similarity s(*u_i_*, *v_j_*)*.* Using these HMMs, the problem of finding the optimal pair of paths in the two networks is translated into that of finding the optimal pair of state sequences in the two HMMs that jointly maximize the probability *P*(**q**, **u**, **v**) of the “virtual observation sequence”:

We can use log *P*(**q**, **u**, **v**) as the alignment score *H*(**u**, **v**), and find the best matching pair of paths using dynamic programming [15]. For this purpose, we first define the score for the most probable pair of paths of length *t*(≤ *L*) as follows:(1)

where *t*_*w*_1__ (*u_i_*, *u_j_*) and *t*_*w*_2__ (*v_k_*, *v_l_*) are the logarithms of transition probabilities determined by the interaction reliability scores in the respective networks. Next, we find the optimal pair of paths (u*, v*)(2)

by iteratively computing the score in (1) for *l* = 1, 2, ⋯ , *L.* Instead of finding only the best matching pair of paths, we can also search for the top *k* path pairs by replacing the max operator in (2) by an operator that finds the *k* largest scores. The computational complexity of the described dynamic programming algorithm is only *O*(*kLM*_1_*M*_2_) for finding the top *k* pairs of matching paths. Note that the computational complexity is linear with respect to each parameter *k*, *L*, *M*_1_, and *M*_2_*.*

In our previous implementation of HMM-based local alignment [14,15,20], we have used the sequence similarity between individual molecules to measure the node similarity *s*(*u_i_*, *v_j_*)*.* As we discussed earlier, it is desirable to integrate all the available information to measure the similarity between network nodes, instead of relying on the similarity between individual molecules. In this paper, we propose to use a semi-Markov random walk model to define a global correspondence scoring scheme for measuring node similarity by incorporating the topological properties around the nodes. As we will demonstrate later, the use of global correspondence scores can improve the accuracy and robustness of the HMM-based alignment results.

### Computation of global correspondence scores through semi-Markov random walk

In order to predict the global correspondence between nodes, we should first consider the similarity between the corresponding molecules themselves, in terms of sequence, structure, and/or function. However, considering that biomolecules carry out their functions through intertwined interactions with other molecules, it is important to consider these interaction patterns as well when evaluating the global similarity between nodes. As recently proposed and discussed in [10,18,21,22], Markov random walk can provide an elegant framework for evaluating the global correspondence between nodes that belong to different networks by seamlessly integrating the similarity between the nodes themselves and that between their interaction patterns.

In this work, we adopt the semi-Markov random walk approach [18] to compute the global correspondence scores for the node similarity *s*(*u_i_*, *v_j_*)*.* The basic idea of this scheme is to perform a simultaneous semi-Markov random walk on  and , such that the random walker moves to one of the neighboring nodes in each network at each time point. The next node is randomly selected among all the neighboring nodes, where nodes with higher interaction reliability have a larger chance to be selected. The time that the random walker spends at a given pair of nodes  and  is proportional to the sequence similarity between the nodes. According to this model, the long-run proportion of time that the random walker spends at (*u_i_*, *v_j_*) will increase if *u_i_* and *v_j_* have higher individual node similarity (e.g., sequence similarity). Furthermore, the proportion of time spent at (*u_i_*,*v_j_*) will also increase if the two nodes are surrounded by similar nodes, hence have a higher topological similarity. As a result, this semi-Markov random walk provides an elegant way of evaluating the global similarity between nodes by integrating individual node similarity and topological similarity. Using this model, we can compute the global correspondence score as follows:(3)

in which *π*_1_(*u_i_*) is the stationary probability of visiting node *u_i_* in an ordinary Markov random walk on , *π*_2_(*v_j_*) is the stationary probability of visiting *v_j_* in a Markov random walk on , and *h*(*u_i_*,*v_j_*) estimates the individual node similarity between *u_i_* and *v_j_*, which is measured in terms of sequence similarity in this work. The above scheme is conceptually similar to the one proposed in [10], where the similarity between two nodes in different networks are measured by *linearly* combining the topological similarity score and the sequence similarity score. The resulting score can be viewed as the long-run proportion of time spent at the given pair of nodes based on a “Markov random walk with restart” model, in which the restart probability has to be chosen in advance to balance the contributions from the interaction similarity and the sequence similarity, typically in an ad-hoc manner. Note that such parameter tuning is not needed in the semi-Markov random walk approach adopted in this work.

In the following sections, we analyze the effect of using the global correspondence scores in the HMM-based local network alignment method. More specifically, we evaluate the performance of the HMM-based local network alignment method when using the global correspondence score for *s*(*u_i_*, *v_j_*) given in (3), and compare it to the performance of the HMM-based alignment method that directly uses the sequence similarity score with *s*(*u_i_*, *v_j_*) = *h*(*u_i_*, *v_j_*), as originally proposed in [14,15].

## Results and discussion

### Aligning synthetic networks

**Figure 1:**
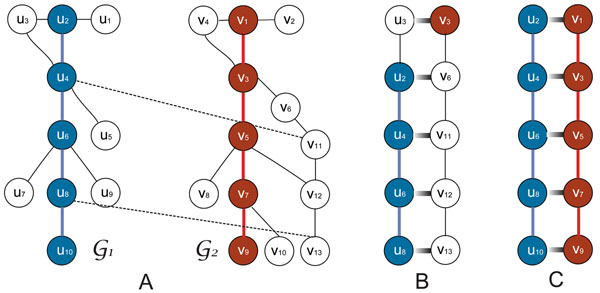
An illustrative example that demonstrates the advantage of using global correspondence scores: (A) Two small synthetic networks that contain similar paths (shown in colors); (B) The top pair of aligned paths predicted by the HMM-based alignment algorithm using the individual node similarity scores; (C) The top pair of aligned paths predicted by the HMM-based method using global node correspondence scores computed by semi-Markov random walk.

To illustrate the advantages of using the global correspondence scores in the HMM-based local network alignment scheme, we first conducted a set of experiments based on synthetically generated networks. Figure 1(A) shows two simple synthetic networks, each of which contains a similar core path, respectively marked in dark blue and dark red. We added more nodes around the core path in each network in a way that the corresponding nodes in the core paths have similar local topological structures. We further assigned individual node similarity scores, where nodes in the respective networks that are located at similar vertical levels were given higher scores. We assigned exceptionally high similarity scores to two node pairs (*u*_4_, *v*_11_) and (*u*_8_,*v*_13_), which are shown by dashed lines in Fig. 1(A). These two highly similar pairs are analogous to molecules in real biological networks that have high sequence similarity without real biological significance. Such nodes can mislead the alignment algorithm, yielding inaccurate alignment results. In fact, we may typically face similar problems when aligning two or more large-scale biological networks. The network adjacency matrices and the node similarity matrix are provided in the *Additional file *1.

We applied the HMM-based local alignment to identify the most similar pair of paths of length *L* = 5. The identified top pair of paths when directly using the assigned node similarity scores is shown in Fig. 1(B). We notice that the alignment result is strongly influenced by the high similarity pairs (*u*_4_,*v*_11_) and ((*u*_8_, *v*_13_) in this case and the prediction does not capture the obvious topological similarity in the two networks. Next, we computed the global correspondence scores between nodes based on the semi-Markov random walk scheme and used these scores in the alignment algorithm, instead of the original node similarity scores. Figure 1(C) shows the top path alignment for this case, where the core paths were accurately identified as we expect based on the topology of the two networks. Simulations based on other small synthetic networks, constructed in similar ways, yielded similar results (see *Additional file *1 for other examples).

For a more thorough performance comparison between the two different schemes—the original scheme that directly uses the individual similarity scores and the proposed scheme that uses the global correspondence scores computed by semi-Markov random walk—we further created a benchmark set that consists of large synthetic networks generated based on a scale-free model [23]. Although we can also evaluate the performance of network alignment algorithms by aligning real biological networks and measuring the accuracy of the alignment results using functional annotations based on Gene Ontology (GO) terms [24] or KEGG ortholog (KO) group annotations [25], these annotations are still highly incomplete and may not accurately reflect the real functional similarity between molecules. As a result, a carefully constructed synthetic benchmark dataset may provide a better benchmark for evaluating future network alignment algorithms.

To construct the synthetic networks, we first randomly generated an undirected seed network  of size 20 with an average degree of 10. Next, we grew this network according to the BA (Barabasi and Albert) model [23] to generate a random scale-free network using the preferential attachment algorithm [26]. In this algorithm, at each time step, a new node is added to the network and connected to *m* existing nodes with a probability that is proportional to the number of links that the nodes already have. As shown in [23], the resulting network captures several important characteristics of real PPI networks. The scale-free degree distribution is one such property, which means that the degree distribution of the network approximately follows the power law *P*(*k*) ~ *k*^*γ*^, where *γ* is the degree exponent. In this work, we used this model with *m* = 10 to grow  to a network of size 1000. Once  was created, we duplicated the network into two identical networks  and *.* To model the functional coherence between orthologous proteins, we then assigned a distinct group annotation to each pair of corresponding proteins in the two networks. More specifically, both the node *u_i_* in  and the node *v_i_* in  were assigned to the *i*th functional group. We randomly assigned individual node similarity scores between orthologous nodes according to the Gaussian distribution  with mean *µ_o_* = 300 and standard deviation *σ_o_* = 100. The node similarity scores between non-orthologous nodes were randomly assigned according to a different distribution , where *σ* = 100, and *µ* was used as a free parameter that determines the level of overlap between the two similarity score distributions. Node similarity scores below a certain threshold (set to 50 in this work) were set to zero. For every node, we also restricted the number of non-orthologous nodes with a nonzero similarity score to 10. These settings were used to make the resulting random networks similar to real PPI networks in public databases.

Up to this point, the two networks  and  were topologically identical. To introduce topological differences in these networks, we randomly deleted 10% of the edges in  and . Furthermore, we also randomly deleted 10% of the nodes in the two networks and added back an identical number of new nodes by growing the networks using the preferential attachment algorithm. No functional group was assigned to these randomly inserted nodes. The node similarity between the inserted nodes in one network and the nodes in the other network was sparsely assigned according to , as before.

**Figure 2:**
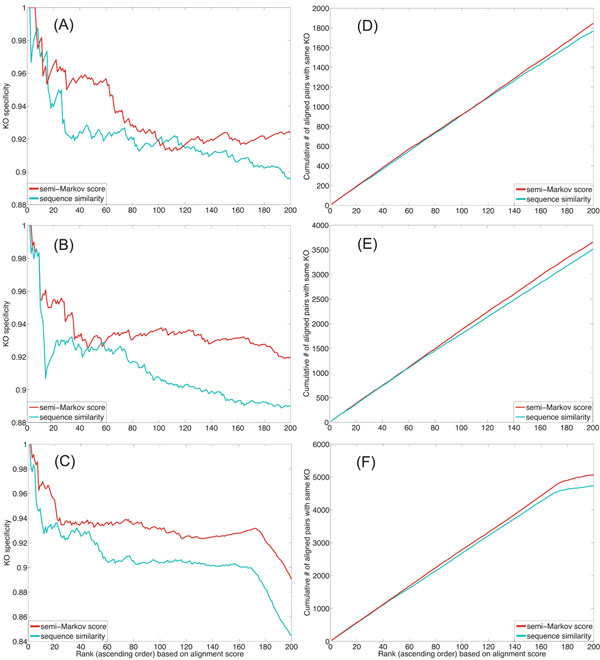
Functional specificity (A,B,C) and coverage (D,E,F) of the synthetic network alignment obtained by the HMM-based local network alignment method using the global node correspondence scores and the individual node similarity scores for *L* = 10 (A,D), *L* = 20 (B,E), and *L* = 30 (C,F).

Based on the above model, we generated two networks  and  with *µ* = 200. Using the HMM-based local network alignment algorithm, we identified the top 200 high-scoring path alignments with gaps. To find the top 200 path alignments, we iterated the following steps: (i) find the optimal path alignment; (ii) store the predicted alignment; (iii) remove the interactions included in the path alignments; (iv) repeat the experiment to find the next path alignment. The accuracy of the identified path pairs are measured based on the group annotations of the aligned nodes as in [11,15]. We define the cumulative specificity of the top *k* alignments as follows:(4)

where  the total number of correctly aligned node pairs in the top *i*th alignment, and  is the total number of annotated node pairs also in the top *i*th alignment. We also define the cumulative coverage of the top *k* alignments as , which computes the cumulative number of pairs with the same functional annotations. This metric measures the size of the accurately aligned network regions covered by the top *k* path alignments. We repeated the alignment experiments for different path lengths: *L* = 10, 20, and 30. The alignment results are summarized in Fig. 2. As we can see in this figure, the use of global node correspondence scores computed by the semi-Markov random walk approach significantly improves the specificity of the alignment, while the coverage is also slightly improved. This clearly shows that incorporating topological information of network nodes into the alignment process can be crucial in obtaining accurate alignment results when the individual node similarity scores are not highly discriminative by themselves.

**Figure 3:**
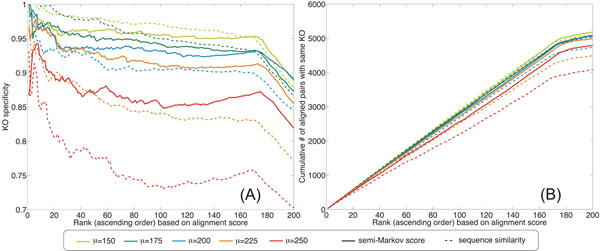
Functional specificity (A) and coverage (B) of the synthetic network alignment obtained using global correspondence scores and individual node similarity scores for various *µ* = {150, 175, 200, 225, 250}.

Next, we investigated performance improvement by the proposed approach for varying levels of overlap between the node similarity score distribution for orthologous nodes and that for non-orthologous nodes. We set *L* = 30 and evaluated the performance of the HMM-based alignment algorithm using the global correspondence scores and the individual similarity scores, respectively, for various values of *µ.* The simulation results are shown Fig. 3. As shown in this figure, the advantage of using the global correspondence scores for network alignment becomes more prominent for larger *µ.* When *µ* is small (e.g., *µ* = 150), we can accurately identify orthologous nodes by using individual node similarity scores. However, as *µ* increases and the individual node similarity scores become less discriminative, it becomes more critical to utilize topological information to identify orthologs. This can be seen when *µ* = 250, in which case the use of global correspondence scores can remarkably improve the accuracy of the alignment. It is also apparent from the figure that the performance of local network alignment using individual node similarity degrades much faster as the individual similarity gets less informative. This implies that we can obtain more robust and reliable alignment results by incorporating the global correspondence scores into the HMM-based local network alignment algorithm. (The URL for downloading the synthetic networks used in our experiments can be found in the *Additional file *1*.*)

### Aligning microbial PPI networks

For further evaluation of the proposed method, we performed pairwise alignments of three microbial PPI networks obtained from [7]. In our experiments, we aligned the *E. coli* network and the *C. crescentus* networks to detect conserved functional modules in the two networks. Similarly, we also performed a pairwise alignment between the *E. coli* and the *S. typhimurium* networks to find conserved modules in these networks. As before, we have assessed the accuracy of the alignment results using two metrics—namely, specificity and coverage—based on the KEGG ortholog (KO) group annotations [25] of the proteins in the microbial networks. A protein alignment is regarded as being correct if the aligned proteins have the same KO group annotations, and incorrect if the annotations do not agree.

In these experiments, the parameters of the HMMs were chosen as follows. First, the transition scores *t_w_*(*u_i_*,*u_j_*) in (1) were determined based on the presence (or absence) of protein interactions in the microbial networks determined by the SRINI algorithm [27]:(5)

Second, we used the BLASTP hit scores between protein pairs, provided in [7], as the individual node similarity scores. The global correspondence scores were computed according to the semi-Markov random walk approach described earlier. These two types of node similarity scores were normalized such that they lie in the same range.

**Figure 4:**
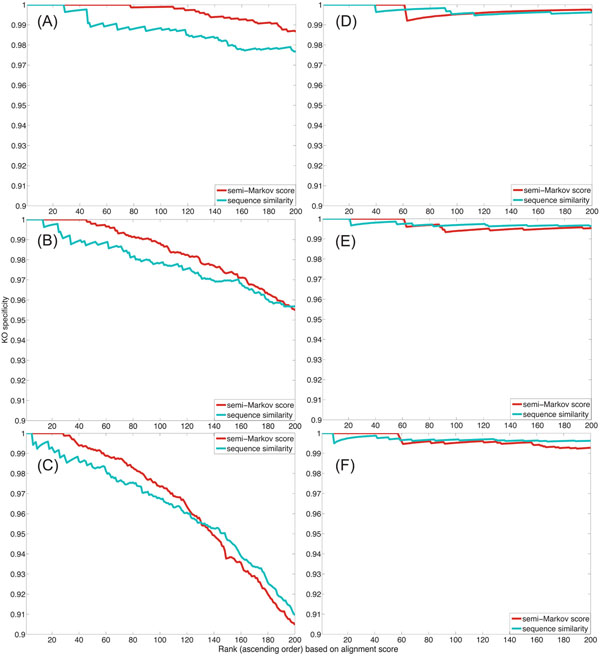
Functional specificity of the microbial network alignment obtained by the HMM-based local network alignment method using the global correspondence scores and the individual node similarity scores. The cumulative specificity of the top 200 path alignments are shown: (A) Pairwise alignment between *E. coli* and *C. crescentus* networks with *L* = 10; (B) Pairwise alignment between *E. coli* and *C. crescentus* networks with *L* = 20; (C) Pairwise alignment between *E. coli* and *C. crescentus* networks with *L* = 30; (D) Pairwise alignment between *E. coli* and *S. typhimurium* networks with *L* = 10; (E) Pairwise alignment between *E. coli* and *S. typhimurium* networks with *L* = 20; (F) Pairwise alignment between *E. coli* and *S. typhimurium* networks with *L* = 30.

**Figure 5:**
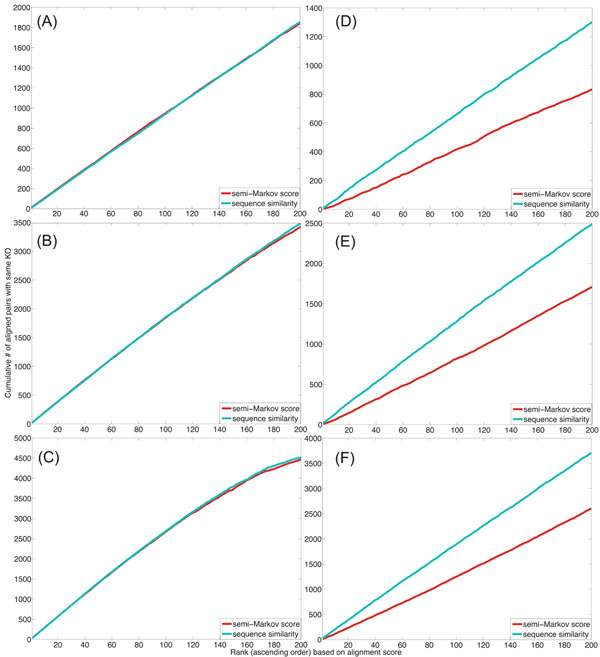
Functional coverage for microbial network alignment using the new semi-Markov node similarity and the original sequence similarity: The cumulative sensitivity of the top 200 aligned pathways obtained from (A) the pairwise alignment between *E. coli* and *C. crescentus* networks with *L* = 10; (B) the pairwise alignment between *E. coli* and *C. crescentus* networks with *L* = 20; (C) the pairwise alignment between *E. coli* and *C. crescentus* networks with *L* = 30; (D) the pairwise alignment between *E. coli* and *S. typhimurium* networks with *L* = 10; (D) the pairwise alignment between *E. coli* and *S. typhimurium* networks with *L* = 20; (D) the pairwise alignment between *E. coli* and *S. typhimurium* networks with *L* = 30.

Based on the constructed HMMs, we used our local network alignment algorithm to find the 200 top-scoring pathway alignments with gaps. This experiment was also repeated for several different virtual path length: *L* = 10, 20, and 30. In all our experiments, we disallowed multiple occurrences of identical protein pairs in a given path alignment. The cumulative specificity *cs_k_* of the pairwise alignment of the *E. coli* and the *C. crescentus* networks using the two different types of node similarities are shown in Figs. 4(A), (B), and (C) for *L* = 10, 20, and 30, respectively. The results of the pairwise alignment of the *E. coli* and the *S. typhimurium* networks are shown in Figs. 4(D), (E), and (F). Figure 5 shows the cumulative coverage *cc_k_* of the predicted path alignments using the two different node similarity scores.

As we can see from the pairwise alignment results of the *E. coli* and the *C. crescentus* networks, shown in Fig. 4(A), (B), (C) and Fig. 5(A), (B), (C), when the coverage of the predicted path alignments is comparable, using the global correspondence scores results in higher specificity compared to using the individual node similarity scores. This implies that HMM-based network alignment based on global correspondence scores can more effectively capture the functional similarity between nodes. However, as we can see in Fig. 5(D), (E), (F), the protein pairs aligned using the semi-Markov random walk based global correspondence scores are less annotated (as reflected in the lower coverage *cc_k_*) for the pairwise alignment of the *E. coli* and the *S. typhimurium* networks, in which case the specificity of the predicted alignment is not necessarily improved by the global scores. This can be seen in Fig. 4(D), (E), (F). One possible explanation for this observation is that the KO group annotations may have been curated largely baed on sequence similarity between proteins. For example, for remote orthologs that do not have high sequence similarity, it may be practically difficult to judge to which KO group they should belong since there is not enough evidence. From this point of view, network alignment using global correspondence scores obtained from semi-Markov random walk could be used to validate and improve functional annotation of proteins.

In order to further examine the biological significance of the alignment results obtained from the proposed method, we retrieved the unannotated protein pairs that are aligned in the top path alignments predicted by comparing the *E. coli* and the *S. typhimurium* networks. As these proteins do not have curated functional annotations, such as GO terms or KO groups, we manually checked the protein information for the first 20 unannotated pairs in the Protein database at the National Center for Biotechnology Information (NCBI) [28]. Table 1 shows the assigned gene names and regions names based on the Genlnfo Identifiers (GIs) of these aligned proteins. Among these 20 protein pairs that are unannotated in the KEGG database, 18 pairs consist of proteins that have been assigned with the exactly same gene names and region names. In fact, these protein pairs indeed have similar cellular functionalities, where many of them are membrane proteins. Further information about these protein pairs can be found at the URLs included in the *Additional file *1*.* For the remaining two protein pairs (shown in bold face in Table 1), we could see that the aligned proteins were not assigned with the same gene names because the proteins in the *S. typhimurium* network were not annotated. When we checked the region names of the aligned proteins in each pair, we could see that the proteins in the first pair have the same region name “Transposase_3l” and those in the second pair have the same region name “DUF1131”. These observations suggest that the HMM-based network alignment method using semi-Markov random walk scores may provide a promising framework for automatic functional annotation of proteins.

**Table 1:** Gene names and Region names based on the GenInfo Identifiers (GIs) of the top 20 unannotated protein pairs that are aligned in the top conserved paths. Synonymous gene names are shown within parentheses.

*E. coli*			*S. typhimurium*		
GI	Gene name	Region name	GI	Gene name	Region name

16131641	wzzE	Wzz	16767194	wzzE	Wzz
49176398	viaA (yieM)	VWA_YIEM_type	39546380	yieM	VWA_YIEM_type
16131399	yhjJ	PqqL	16766899	yhjJ	PqqL
16131130	aaeB (yhcP)	FUSC	16766659	yhcP	FUSC
16130240	**yfcl**	**Transposase_3**1	16767050	**STM3766**	**Transposase_31**
49176226	bamC (nlpB)	Lipoprotein_l8	16765808	nlpB	Lipoprotein_l8
16129342	ydbH	DctA-YdbH	16764990	ydbH	DctA-YdbH
49176233	sseB	SseB	16765855	sseB	SseB
16129572	ydgA	PRK11367	16764812	ydgA	PRK11367
16131557	yidR	propeller_TolB	16767096	yidR	TolB
16131404	bcsB (yhjN)	BcsB	16766904	yhjN	BcsB
16130950	ygiF	CYTH-like_Pase_CHAD	16766502	ygiF	CYTH-like_Pase_CHAD
16131855	yjbH	DUF94O	16767475	yjbH	DUF940
16128005	yaaW (htgA)	Ubiq_cyt_C_chap	16763400	htgA	Ubiq_cyt_C_chap
16130391	ypfG	DUF1176	16765796	ypfG	DUF1176
16130357	**yfeY**	**DUF1131**	16765767	**STM2447**	**DUF1131**
49176330	yhdP	PRK10899	16766664	yhdP	PRK10899
16131526	yicH	AsmA	16767033	yicH	AsmA
16131275	yrfF	IgaA	16766783	yrfF	IgaA
16129282	ycjx	DUF463	16765028	ycjx	DUF463

## Conclusion

In this paper, we studied the effect of using a global similarity scoring scheme to measure the node similarity and incorporating these global scores in the HMM-based local network alignment algorithm. We used the semi-Markov random walk framework to compute the global correspondence scores between nodes in different networks. The resulting scores can effectively combine the topological similarity of the subnetworks around the network nodes as well as their individual molecular similarity. Experimental results on microbial protein-protein interaction networks and synthetic scale-free networks show that the use of global correspondence scores can better identify paths with similar topological properties, thereby improving the specificity of the predicted alignment. We believe that the proposed alignment scheme can provide an effective and computationally efficient framework for developing robust and accurate functional annotation tools for proteins.

## Authors contributions

Conceived and designed the experiments: XQ, SMES, BJY. Performed the network alignment experiments: XQ. Implemented the semi-Markov random walk based scoring scheme: SMES. Analyzed the data and wrote the paper: XQ, SMES, BJY.

## Supplementary Material

Additional file 1 **supplement.pdf** This file contains: (i) information of the benchmark set that contains randomly generated synthetic networks, (ii) additional experimental results, and (iii) relevant information regarding the unannotated pairs of aligned proteins in microbial networks. Click here for file
