# Supplementary material for: Enhancing the accuracy of HMM-based conserved pathway prediction using global correspondence scores
Source: BMC Bioinformatics. 2011 Oct 18;12(Suppl 10):S6. doi: 10.1186/1471-2105-12-S10-S6 (PMC3236849; doi:10.1186/1471-2105-12-S10-S6)
Supplement: Additional file 1 — supplement.pdf This file contains: (i) information of the benchmark set that contains randomly generated synthetic networks, (ii) additional experimental results, and (iii) relevant information regarding the unannotated pairs of aligned proteins in microbial networks. [file 1471-2105-12-S10-S6-S1.pdf]

This document contains supplementary materials for the manuscript **"Enhancing the accuracy of HMM-based conserved pathway prediction using global correspondence scores"**, and includes: (i) information of the benchmark set that contains randomly generated synthetic networks, (ii) additional experimental results, and (iii) relevant information regarding the unannotated pairs of aligned proteins in microbial networks.

* **Note:** The order of the references in this document is *not* identical to the order in the manuscript. The list of references cited in this file can be found on the last page.

Synthetic networks

Performance comparison for small illustrative synthetic networks

In addition to the alignment examples of synthetic networks shown in the manuscript, we also tried a few other examples, one of which is shown in Fig. 1. The network adjacency matrices, the originally assigned individual node similarity matrices, and the MATLAB code to compute the semi-Markov random walk based global correspondence scores can be found in the following Web page at `http://www.cse.usf.edu/~xqian/smrw/`.

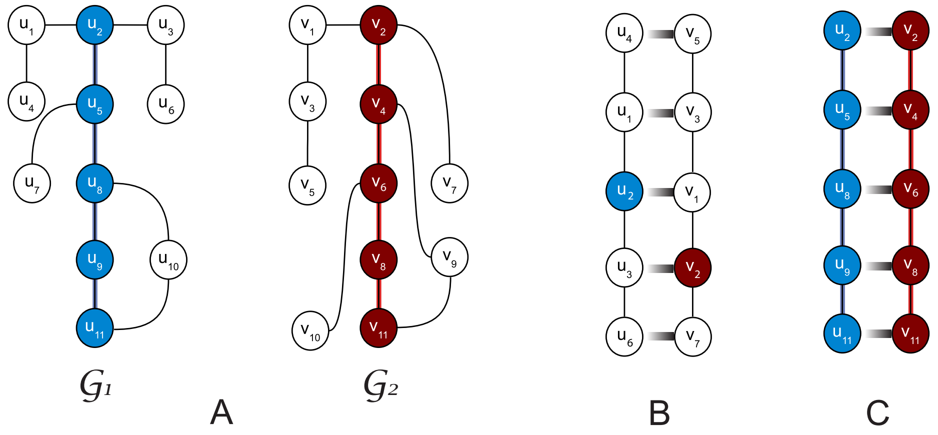

Figure 1: An additional example of why global correspondence scores are better: (A) Two small synthetic networks with essential path components marked in colors; (B) The top pair of aligned paths by HMM-based alignment using individual node similarity; (C) The top pair of aligned paths by HMM-based alignment using semi-Markov random walk based global correspondence scores.

Benchmark set of randomly generated synthetic networks

As described in the manuscript, we have randomly generated a set of scale-free networks for performance evaluation of the proposed network alignment scheme. These networks can be found at:
`http://www.cse.usf.edu/~xqian/smrw/scale_free_random_networks/`.

Microbial networks

We obtained the microbial networks and their KO annotations from Græmlin [Flannick *et al.*, 2006] and KEGG [Kanehisa and Goto, 2000]. The protein IDs of the unannotated pairs of aligned proteins (in the top 200 high scoring similar paths from the *E. coli* and the *S. typhimurium* network pair) can be found in `unannotated_smrw.mat` at `http://www.cse.usf.edu/~xqian/smrw/microbial/`. Further information about the top 20 unannotated pairs of aligned proteins can be found in the Protein database at the National Center for Biotechnology Information (NCBI). URLs to the respective protein information Web pages can be found in `unannotated_top20pairs.txt` at `http://www.cse.usf.edu/~xqian/smrw/microbial/`.

References

[Flannick *et al.*, 2006] Flannick J, Novak A, Srinivasan B, McAdams H, Batzoglou S (2006) Græmlin: General and robust alignment of multiple large interaction networks. *Genome Res* 2006, **16**(9):1169-1181.

[Kanehisa and Goto, 2000] Kanehisa M and Goto S (2000) KEGG: Kyoto encyclopedia of genes and genomes. *Nucleic Acids Res*, **28**:27-30.
